# An Extraordinary Case of Prosthetic Joint Infection by Listeria monocytogenes and Histoplasma capsulatum

**DOI:** 10.7759/cureus.76901

**Published:** 2025-01-04

**Authors:** Tyler Luu, Lauren Daddi, Marwan Azar, Paul Trubin, Ilda Molloy, Anne Spichler-Moffarah

**Affiliations:** 1 Infectious Disease, Yale School of Medicine, New Haven, USA; 2 Internal Medicine, Yale School of Medicine, New Haven, USA; 3 Infectious Disease, Yale New Haven Hospital, New Haven, USA; 4 Orthopedics, Yale School of Medicine, New Haven, USA

**Keywords:** co-infection, histoplasma capsulatum, immunocompromised host, listeria monocytogenes, prosthetic joint infection

## Abstract

Prosthetic joint infections (PJIs) are serious complications of hip and knee arthroplasty. Gram-positive organisms are the most common etiology of PJIs. Less common bacteria and certain fungi may also present as culprits on rare occasions. *Listeria monocytogenes *and* Histoplasma capsulatum *are unique etiologies of PJIs. Co-infection with both organisms is even more extraordinary. We present a case of an immunocompromised host with a remote history of right knee arthroplasty who presented with late PJI. Synovial fluid culture revealed *Listeria monocytogenes* and *Histoplasma capsulatum* co-infection. Both of these pathogens can form biofilm; therefore, a two-stage exchange surgery is recommended, in addition to antimicrobial treatment. Although these organisms were not expected, with certain epidemiologic risk factors and hosts' conditions, clinicians should always remain vigilant.

## Introduction

Prosthetic joint infection (PJI) is a challenging complication of joint arthroplasties, involving a complex interplay between microbes and the host immune response [1]. *Listeria monocytogenes* accounts for ~2% of hip and knee PJIs [2,3], while fungal PJIs, primarily *Candida* species, represent less than 1% of all cases. *Histoplasma capsulatum *causing PJI is extremely rare, with as few as seven cases reported in the literature [4,5].

The host immune response plays an important role in the pathogenesis of PJI with important management implications [6]. General risk factors include age, conditions of altered immunity such as rheumatoid arthritis (RA), and iatrogenic immunosuppression [1,6]. Bone and joint listeriosis risk increases with age above 60 years, the presence of foreign material, and cirrhosis, among others [2]. *Histoplasma* PJI risk factors are less defined, often involving endemic exposure or an immunocompromised state (i.e., tumor necrosis factor (TNF)-alpha inhibitors and/or steroids), though cases without these factors exist [5,7].

Polymicrobial PJIs (25%) are less common than monomicrobial PJIs (70%) and rarer in late PJIs (20%) [8,9]. Late-onset PJI, occurring 12-24 months after surgery, often results from hematogenous spread or an indolent infection acquired at the time of surgery. In terms of clinical presentation, late PJI usually presents with pain, sinus tract, and difficulty with mobility. *Listeria* PJIs are typically subacute, paucisymptomatic, and monoarticular. The treatment combines surgery and antimicrobials, though limited clinical experience with *Listeria* and *Histoplasma* PJIs leads to varied management approaches in the literature. *Listeria* PJI generally has favorable outcomes [2,3]. *Histoplasma* PJI, often isolated with delayed culture growth, responds well when managed with appropriate antifungal and resection arthroplasty [5]. While each of these infections is independently quite rare, co-infection involving both has yet to be described in the literature. This article presents the first reported case of *Listeria monocytogenes *and *Histoplasma capsulatum* co-infection of a prosthetic knee joint in an immunocompromised host.

## Case presentation

A 71-year-old woman from Ecuador was admitted to a tertiary academic medical center with a chief concern of acute-on-chronic right knee pain in the setting of total knee replacement 14 years prior in Ecuador. She experienced a similar episode four months prior treated with therapeutic intra-articular aspiration in Ecuador, with no records from aspiration results. This time, the patient had experienced symptoms for >4 weeks prior to presentation to the emergency department. The patient was born and raised in Quito, Ecuador, where she resided and presented while visiting family in the United States. Pertinent past medical history was notable for RA treated with prednisone 10 mg daily and methotrexate 12.5 mg weekly in Ecuador. There had not been any changes to her immunosuppressants prior to this episode. While in Ecuador, she reportedly had consumed unpasteurized queso fresco.

On admission, hemodynamic stability was noted. Physical examination was notable for diffuse swelling of the right knee, particularly at the medial aspect of the joint, with associated warmth and mild overlying erythema. Significant range of motion restriction of the right knee was noted due to tenderness and edema. Complete blood count with differential showed acute, neutrophil-predominant leukocytosis to 18.9 × 1000/µL (normal range 4.0-11.0 × 1000/µL). C-reactive protein was markedly elevated to 220 mg/L (normal range <10 mg/L).

The patient underwent right knee fluid aspiration yielding turbid fluid. Synovial fluid analysis was notable for 113,000 cells/µL nucleated consisting of 96% neutrophils (normal range <200 cells/µL). Fluid was sent for bacterial culture which yielded 1+ *Listeria monocytogenes* after 27 hours of incubation, while the fungal culture remained incubated.

**Figure 1:**
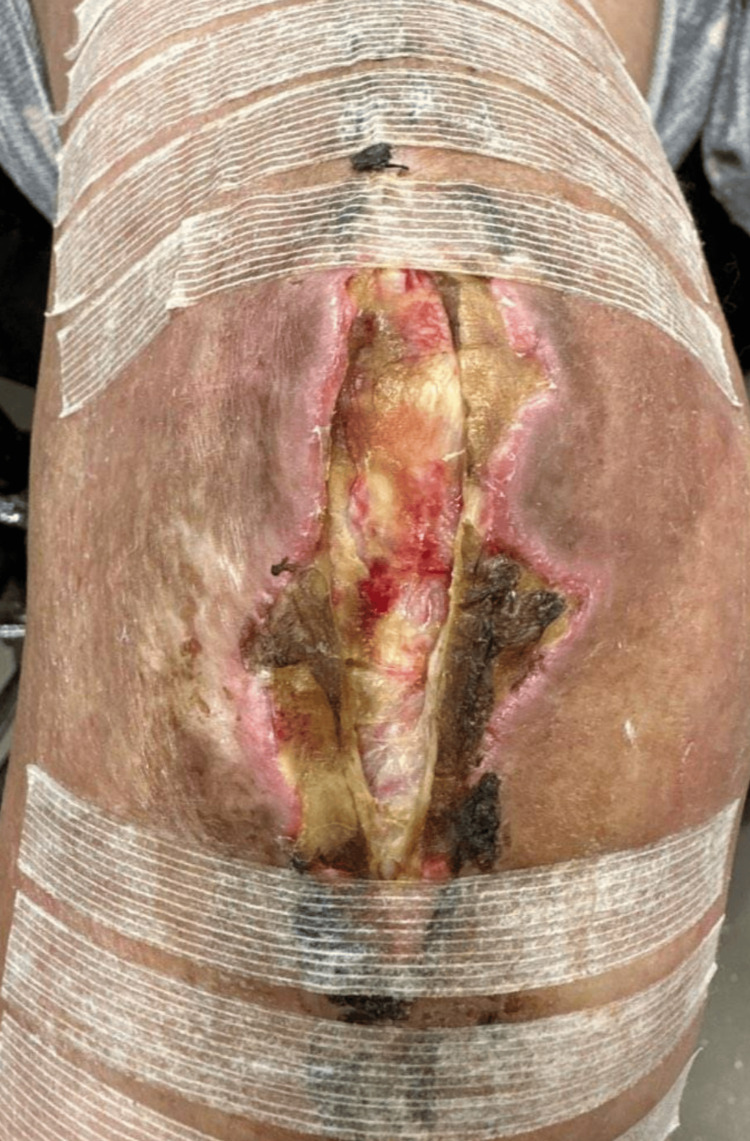
Right knee showed full-thickness dehiscence with surrounding dry yellow eschar about the patella

The patient rapidly developed septic shock on the second day of hospitalization, requiring the initiation of vasopressors. Given the chronicity of symptoms for at least four months and deteriorating clinical presentation, the patient underwent surgical intervention with right knee debridement, irrigation, explantation of hardware, and placement of an articulating antibiotic spacer. Intraoperative findings included intracapsular purulent material of milky-white, loculated consistency and yellow purulence tracking up the medal aspect of her thigh and down the patient's calf. Intraoperative tissue cultures grew *Listeria monocytogenes*, and she was discharged with six weeks of intravenous (IV) ampicillin 2 g every four hours. Upon one-month postoperative follow-up, symptoms had worsened despite the antibiotic treatment with increasing pain, swelling, drainage, and wound dehiscence at the surgical site (Figure 1).

Subsequently, 2 colony-forming units (CFUs) of *Histoplasma capsulatum* were retrieved from the initial synovial aspiration fungal media. *Histoplasma* urine antigen, however, returned negative at <0.2 ng/mL (reference range <0.2 ng/mL).

With clinical deterioration and only limited soft tissue coverage, the patient underwent staged orthopedic and plastic surgery procedures with repeat debridement, placement of static antibiotic spacer, and subsequent soft tissue anterolateral thigh flap. Multiple perioperative bacterial, fungal, and acid-fast bacillus (AFB) cultures were obtained, from which only bacterial cultures grew *Listeria monocytogenes*.

**Figure 2:**
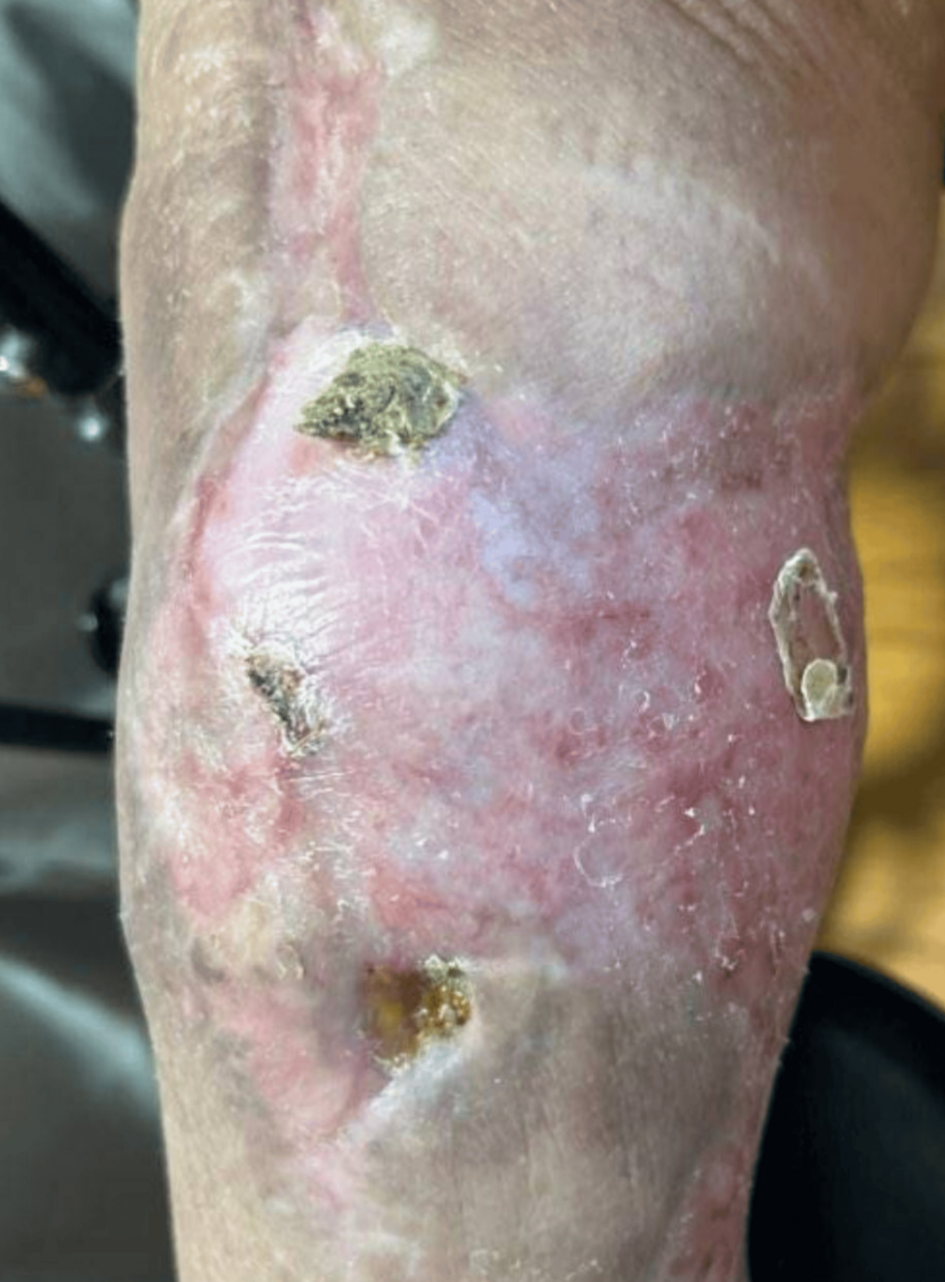
Right knee demonstrated healthy, well-healing granulation tissues without signs of acute infection

The patient was treated with IV ampicillin 2 g every four hours and oral solution itraconazole 200 mg twice daily. Her postoperative course was complicated by acute kidney injury secondary to acute interstitial nephritis, which resolved upon the discontinuation of the ampicillin (eight weeks after initiation). Co-trimoxazole 800/160 mg two tablets every 12 hours was utilized to target listeriosis for an additional six weeks. She ultimately completed a six-month course of itraconazole for her histoplasmosis. For her RA, hydroxychloroquine was started in lieu of methotrexate, while the prednisone dose was reduced from 10 mg to 5 mg daily in the setting of active infection. At her most recent follow-up visit (10 months since her diagnosis), the patient's right knee exhibited well-healing scars, without signs of acute or recurrent infection (Figure 2).

## Discussion

PJIs are serious complications of hip and knee arthroplasty and commonly require revision arthroplasty. They are characterized by a complex and intricate interplay between microbes, predominantly bacteria but occasionally fungi, and the host immune response. *Listeria monocytogenes* and *Histoplasma capsulatum*, however, are only rarely considered pathogenic PJI organisms. Due to their distinctive virulence in PJI, the management and treatment of *Listeria monocytogenes* and *Histoplasma capsulatum* PJI require specific approaches.

*Listeria monocytogenes*, primarily a food-borne pathogen, enters via the gastrointestinal tract. Due to *Listeria monocytogenes* causing facultatively intracellular infection, cell-mediated immunity is the main defense mechanism. Conditions or medications altering cell-mediated immunity may predispose to listeriosis [3]. Unlike native joint infections, PJI involves biofilm formation. Hutchins et al. presented a case of recurrent *Listeria monocytogenes* PJI following the debridement and retention of a prosthetic hip in which clinical isolates exhibited 41 genes overexpressed in biofilm state as determined via transcriptomics. The authors therefore hypothesized that *Listeria monocytogenes* formed biofilms on the prosthetic joint materials permitting survival and growth with minimal exposure to stressors [10]. For that reason, a two-stage exchange surgery, which consists of debridement and joint prosthetic removal followed by the implantation of a new prosthesis once the infection has cleared with antimicrobial therapy, is recommended to maximize eradication success [5,11].

Ampicillin remains the drug of choice for listeriosis due to its bactericidal activity and limited adverse effect profile, with co-trimoxazole as an alternative for penicillin-allergic patients. Other agents, including meropenem, levofloxacin, linezolid, and vancomycin, are used for regimen simplification or polymicrobial infections. Retained prosthesis may warrant long-term suppression as in this case. Our patient continued to receive suppressive levofloxacin, as a simplified regimen, after completing a six-week treatment course. 

*Histoplasma capsulatum* is a dimorphic fungus endemic to the Ohio and Mississippi River valleys of the United States and certain regions around the world, including Ecuador. While pulmonary infection is the main effect of histoplasmosis, extrapulmonary compartments of involvement may include the gastrointestinal tract, liver, brain, spleen, and bone marrow in immunocompromised hosts. Histoplasmosis, however, rarely causes osteomyelitis or septic arthritis and even less commonly involves joint prosthesis [12]. Similar to *Listeria monocytogenes* infection, functional T cells are critical to host defense. Diagnostically, the sensitivity of a *Histoplasma* enzyme immunoassay (EIA) urine antigen test has been studied to be below 70% in people living with HIV [13]. There are limited data on those without HIV infections. The antifungal treatment of histoplasmosis depends on the immune status and disease severity. Localized joint infection without evidence of multiorgan involvement can be managed with oral itraconazole alone, although the presence of yeast in the joint may imply dissemination. Like *Listeria monocytogenes*, *Histoplasma capsulatum* also has a high potential for biofilm formation; therefore, a two-stage exchange surgery is recommended [11]. The duration of PJI treatment due to *Histoplasma capsulatum* is not clear; however, some cases were treated with more than seven months of antifungal therapy after resection arthroplasty [6]. It is worth noting that in our case, *Histoplasma capsulatum *only grew in small quantity from synovial fluid culture and not from intraoperative tissue cultures (taken prior to starting itraconazole). The question of contamination was considered, but a laboratory investigation did not identify supporting evidence. However, due to the patient's many aforementioned *Histoplasma capsulatum*-specific risk factors, the sterile source of the cultures, and the pathogenic potential of the fungus, the decision was made to treat.

Upon the most recent follow-up, the patient's right knee exhibited minimal bleeding without purulent drainage and mild tenderness with movement. She continued to participate in an outpatient physical therapy program with near-baseline functionality of her right lower extremity.

## Conclusions

*Histoplasma capsulatum* and *Listeria monocytogenes* are rare causes of PJI. Co-infection of these two is even more remarkable. The common predisposed factor is altering cell-mediated immunity, commonly seen in recipients of immunomodulatory therapies. We hypothesize that our patient's impaired immune response and specific epidemiologic risks played an important role in predisposing her to this extraordinary co-infection. Both of these pathogens have a great tendency for biofilm formation which dictates prospective management. Two-stage exchange surgery is the main recommendation, in addition to antimicrobial treatment. The duration of antimicrobial therapy to target the causative pathogens is still under discussion; however, prolonged treatment is preferred. From this particular case, we also appreciate the importance of a multidisciplinary approach from various medical, surgical, and ancillary specialties in managing rare PJIs to ensure good outcomes. It is crucial for healthcare providers to be aware of unusual causative organisms to provide appropriate management.
